# Extracellular Vesicle-Enhanced Stem Cell Therapy in Acute Myocardial Infarction: A Case Report of Cardiac Regeneration from a Bypass Surgery

**DOI:** 10.1007/s12015-025-10910-y

**Published:** 2025-06-12

**Authors:** Halil Hüzmeli, Ayberk Akat, Ahmet Özkara, Emel Ceylan, Ebru Özenç, Erdal Karaöz

**Affiliations:** 1 Department of Cardiovascular Surgery, Liv Hospital Ulus, Istanbul, Turkey; 2 Department of Medical Biology, Faculty of Medicine, Girne American University, Karmi Campus, P.Box: 5, Kyrenia, Cyprus; 3 Department of Nuclear Medicine, Faculty of Medicine, Istinye University, Istanbul, Turkey; 4 Department of Cardiology, Liv Hospital Ulus, Istanbul, Turkey; 5 Faculty of Medicine, Istinye University, Istanbul, Turkey

**Keywords:** Stem cell therapy, Extracellular vesicle therapy, Acute myocardial infarction, Coronary artery bypass graft, Cardiac regeneration

## Abstract

Myocardial infarction is still a significant cause of morbidity and mortality. Coronary artery obstruction reduces blood flow and oxygen supply to the heart muscle, resulting in ischemia and necrosis. Due to the heart's limited healing mechanisms, regenerative therapies to restore cardiac function are being investigated. This case report, describes the utilization of mesenchymal stem cells and extracellular vesicles derived from these cells during coronary artery bypass grafting surgery for the patient who had a recent acute myocardial infarction. A direct injection into the myocardium was performed during surgery after a failed percutaneous coronary intervention. During the follow-up, the patient demonstrated improvements in cardiac function, with the ejection fraction increasing from 28 to 35% as measured by myocardial perfusion scintigraphy, and up to 43% on echocardiographic assessment at six months post-operation, as well as decreases in end-diastolic and end-systolic volumes. Significantly, these advantages remained despite the blockage of the bypass graft. The present case shows that extracellular vesicle-enhanced stem cell treatment may be used in surgical revascularization to restore myocardium in severe ischemic damage.

**Graphical Abstract:**

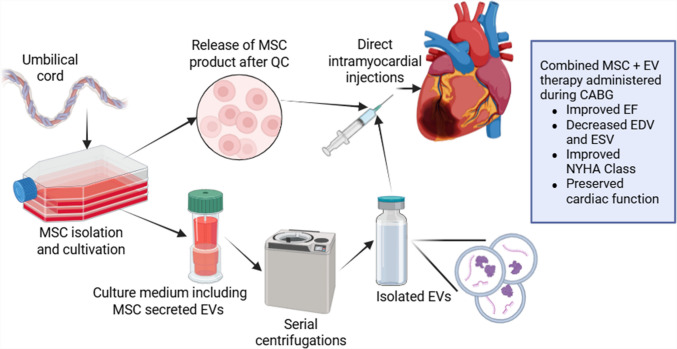

## Introduction

Myocardial infarction (MI) remains a leading cause of morbidity and mortality worldwide. It occurs due to the occlusion of coronary arteries, resulting in ischemia and subsequent necrosis of myocardial tissue [1]. Despite advancements in reperfusion strategies such as percutaneous coronary intervention (PCI) and thrombolysis, many patients experience progressive heart failure (HF) due to the irreversible loss of cardiomyocytes and the limited regenerative capacity of the adult myocardium [2]. Following a myocardial infarction (MI), the heart undergoes an intricate healing process characterized by inflammation, fibrosis, and remodeling. Although these mechanisms are essential for immediate survival, they often lead to adverse remodeling that is characterized by ventricular enlargement and decreased contractility. The constraints of the heart's intrinsic healing systems have spurred the investigation of regenerative treatments with the goal of restoring cardiac function [3].

Regenerative therapies using stem cells are at the forefront of research and clinical applications in cardiovascular disorders. These therapies aim to restore the function of damaged heart tissues, primarily through the regeneration and repair of myocardial cells. Stem cells facilitate this process by replacing damaged cells via transdifferentiation and by secreting factors including growth factors, cytokines, and extracellular vesicles that stimulate anti-inflammatory and anti-apoptotic responses, promoting angiogenesis and immune-stimulation in the microenvironment [4].

Mesenchymal stem cells (MSCs) are among the most studied and utilized in clinical settings due to their ability to differentiate into various cell types and their potent paracrine effects. Among MSCs, those derived from the human umbilical cords (hUC-MSCs) are particularly promising due to their high proliferative capacity and low immunogenicity [4]. Extracellular vesicles (EVs), which have emerged as a novel and safer alternative in regenerative medicine, have recently gained attention. EVs have been recognized as key mediators of intercellular communication, capable of delivering proteins, lipids, and nucleic acids to target cells. These vesicles have shown potential in promoting cardiac repair by enhancing cell survival, reducing fibrosis, and stimulating angiogenesis. EV-based therapies are particularly appealing due to their lower immunogenicity and ability to exert therapeutic effects without the risks associated with direct stem cell transplantation [5].

In this case report, we present a unique clinical application of allogeneic hUC-MSCs combined with EVs delivered concurrently during a coronary artery bypass graft (CABG) procedure. This approach aimed to promote cardiac healing in an acute myocardial infarction (AMI) patient who could not be successfully revascularized through conventional PCI. Our findings underscore the substantial regenerative benefits of synergistically combining stem cells and EVs in the treatment of severe myocardial injury.

## Case Presentation

### Patient History and Initial Presentation

A 48-year-old male patient presented to the emergency department with acute anterior myocardial infarction (MI) at an acute coronary syndrome clinic. His medical history included diabetes mellitus and symptoms consistent with New York Heart Association (NYHA) class III HF. Coronary angiography revealed a complete occlusion of the left anterior descending artery (LAD) and the right coronary artery (RCA). Because of the failed percutaneous coronary intervention (PCI), the medical team decided to proceed with coronary artery bypass grafting (CABG).

### Diagnostic Findings (Pre-Intervention)

The electrocardiogram (ECG) showed ST-segment elevation in the anterior leads (V1-V4), indicating an acute anterior MI. Blood tests indicated elevated levels of troponin I, and indicating damage to the myocardial muscle. A coronary angiography was conducted, which revealed total occlusion of LAD and RCA. Attempts to recanalize the LAD via PCI failed, requiring the use of an alternative treatment approach.

**Fig. 1:**
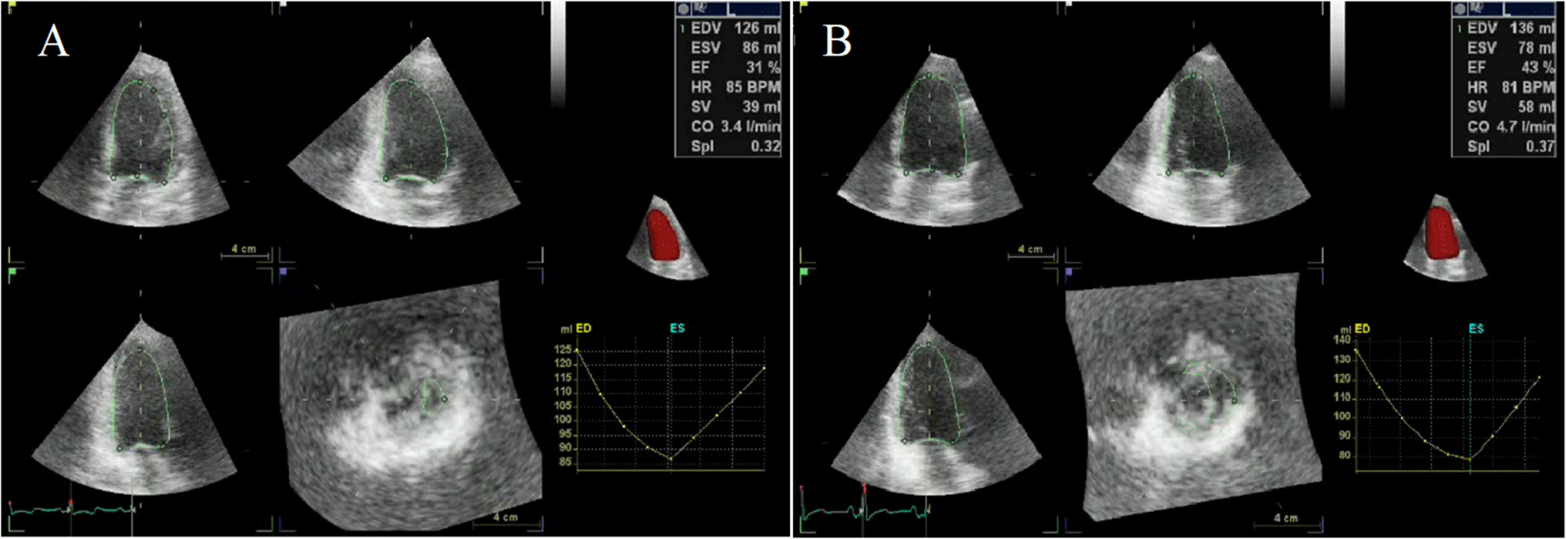
ECHO examination results before and after following MSC + EV therapy. **A** Before surgery: Baseline ECHO examination showing impaired left ventricular function with an EF of 31%, EDV of 126 ml, and ESV of 86 ml. These values indicate significant left ventricular dysfunction, with reduced contractility and cardiac output. **B** Post-op 6 months: Follow-up ECHO at six months post- MSC + EV therapy demonstrates marked improvement in cardiac function, with an EF increased to 43%, EDV to 136 ml, and ESV reduced to 78 ml. The improvement in EF reflects enhanced myocardial contractility, while the reduction in ESV indicates more effective blood ejection during systole. The overall normalization of these parameters suggests a successful therapeutic response, with improved left ventricular performance

The pre-operative transthoracic echocardiogram (ECHO) revealed a severely reduced ejection fraction (EF) of 31%, which was indication of a severe decrease in the left ventricular systolic function. The end-diastolic volume (EDV) was 126 ml, whereas the end-systolic volume (ESV) was measured as 86 ml. These values indicate inadequate ventricular function. ECHO showed akinesia of the anterior, anteroseptal, and anterolateral walls, as well as hypokinesia of the inferior wall. This reflects the extensive myocardial infarction in the LAD territory (Fig. 1).

**Fig. 2:**
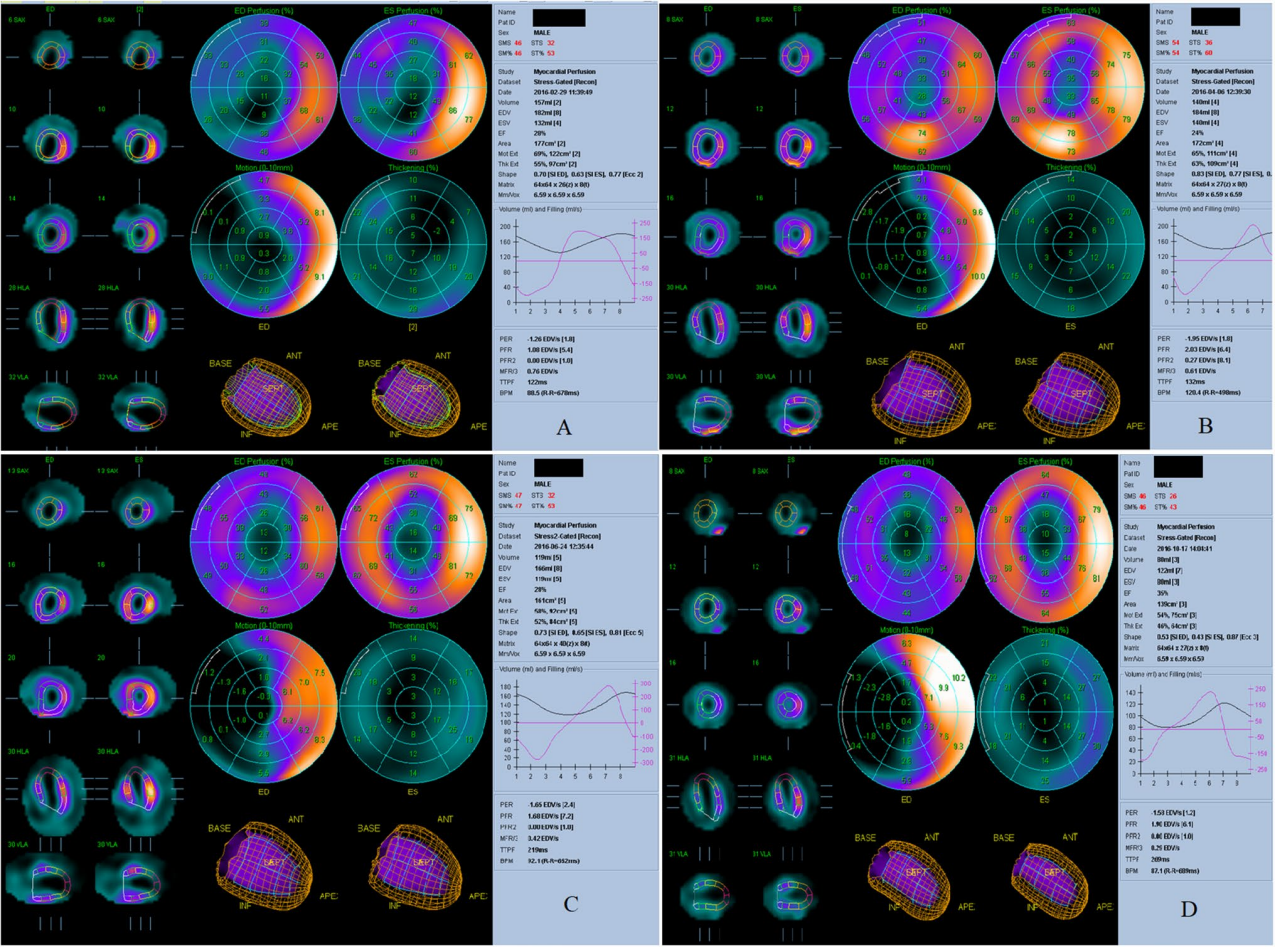
Changes in myocardial perfusion and cardiac function over time following MSC + EV therapy. Polar map views of myocardial perfusion (ED Perfusion %, ES Perfusion %), motion (Motion 0–10 mm), and thickening (Thickening %) along with cross-sectional slices of the heart at four different time points: **A** Initial Evaluation (Pre-op 1 month): Baseline assessment prior to MSC therapy, showing compromised cardiac function with an EF of 28%, EDV of 182 ml, and ESV of 132 ml. These values indicate significant left ventricular dysfunction and impaired myocardial performance. **B** Post-op 1 month: Following MSC therapy, no immediate improvement is observed, with the EF decreasing slightly to 24%, EDV increasing to 184 ml, and ESV rising to 140 ml. **C** Post-op 3 months: Significant recovery begins to manifest, with EF increasing to 28%, EDV decreasing to 166 ml, and ESV reducing to 119 ml. This indicates a gradual but steady improvement in cardiac efficiency and myocardial recovery. **D** Post-op 6 months: Sustained enhancement in cardiac function is noted, with the EF increasing further to 35%, EDV decreasing to 122 ml, and ESV dropping to 80 ml. These results reflect the sustained and progressive benefits of MSC therapy on myocardial function and structure

The analysis of Myocardial Perfusion Scintigraphy (MPS) confirmed the presence of extensive perfusion abnormalities in the anterior, anteroseptal, inferoseptal, and inferior areas, which were suggestive of a recent anterior MI and a prior inferior MI. The results were consistent with ECG and ECHO findings. MPS also indicated an EF of 28%, providing further evidence to support the severity of the left ventricular failure shown on the ECHO (Fig. 2). Due to the severity of myocardial injury and the ineffectiveness of PCI in restoring sufficient blood flow, the patient was considered as a suitable candidate for CABG. Simultaneously, it was proposed to deliver hUC-MSCs together with EVs directly into the heart during the surgical operation, aiming to promote cardiac regeneration and improve cardiac function.

### The hUC-MSC and EV Products

#### Ethics and Consent

hUC-MSC and EV products were acquired from the LivMedCell Good Manufacturing Practice (GMP) facility located in Istanbul, Turkey. The umbilical cord was acquired from a single donor following their informed permission, as authorized by the institutional regulatory board (LivMedCell). The umbilical cord collected after childbirth was sourced from a donor who had completed a full-term pregnancy.

#### Isolation, Production and Quality Control

The umbilical cord obtained from a healthy mother with informed consent was cleansed using phosphate-buffered saline (Invitrogen/Gibco, Paisley, UK). Following vascular excision, the tissue was dissected into explants measuring 5–10 mm^3^. The tissue explants were placed in culture plates and incubated under culture conditions (5% CO_2_ and 37 °C) until the cells migrated out from the explants. The cells were harvested once they reached a confluency of 70–80%, and characterization tests of the cells were carried out at passage 3. The final MSC product consisted of 33 × 10^6^ allogeneic hUC-MSCs, with a cell viability of 94%. Flow cytometric analysis demonstrated the expression of CD73 (99.08%), CD90 (99.3%), and CD105 (92.34%), while negative for HLA-DR (0.08%), CD34 (0.08%), and CD45 (0.08%), consistent with ISCT criteria of MSCs. Quality control assays confirmed sterility and mycoplasma negativity (PCR-based), endotoxin level below 0.25 EU/mL (LAL assay), expressed gene profile consistent with MSC phenotype demonstration, as well as low relative telomerase activity indicating the safety and the non tumorigenicity potential of the product. 33 × 10^6^ allogeneic hUC-MSCs were released from the facility on the operation day. To prepare the EV product, hUC-MSCs were cultured until they reached 90% confluency. The cell media were replaced with serum-free MSC NutriStem® XF Media and incubated in a humidified environment for a minimum of 48 h. After the incubation period, the media containing the cells/cell debris was collected and centrifuged at 300 × g for 5 min, followed by centrifugation at 1000 × g for 10 min to pellet the cells and eliminate any remaining debris. Next, centrifugation was carried out at 5000 × g for 20 min to remove nuclei and dead cell debris. Subsequently, the clarified supernatant underwent ultracentrifugation at a force of 100,000 × g for the duration of 70 min in order to enrich the concentration of the EVs. The pellet was reconstituted in 500 µl of DPBS, with a pH of 7.4, and stored at a temperature of −86 °C until it was utilized. EVs were phenotyped with tetraspanin markers (CD81, CD9, and CD63) and analyzed by flow cytometry (CD9 + 82.3%, CD63 + 86.8% and CD81 + 90%). Nanoparticle tracking analyses (NTA) were also conducted on the final EV product, indicating a size distribution that peaked mostly between 30 to 150 nm. For clinical applications, 5 × 10^9^ particles in 500 µl were used.

### Intervention

The patient had a CABG × 4 operation, conducted without the use of cardiopulmonary bypass. This approach was chosen to minimize the potential risks associated with bypass, particularly in a patient with pre-existing conditions such as diabetes and HF. An intra-aortic balloon pump (IABP) was used for hemodynamic support during the procedure. This device permitted adequate coronary perfusion and lowered the need of myocardial oxygen.

Considering the extent of heart muscle necrosis and the poor regenerative capacity of the heart, the surgical team decided to combine a regenerative approach with the CABG technique. We chose to use hUC-MSCs and EVs derived from hUC-MSCs as they are known to enhance heart muscle healing, reduce inflammation, and promote the growth of new blood vessels [4, 5]. During the operation, a total of 33 × 10^6^ allogeneic hUC-MSCs and 5 × 10^9^ particles of hUC-MSC-derived EVs were precisely injected into the myocardium. The injections were strategically targeted at the anterior, inferior, and septal walls, areas most affected by ischemia. The rationale adopting direct intramyocardial injections was driven by the need to deliver the therapeutic agents precisely to the areas of greatest need, maximizing the potential for localized repair and regeneration. The process included meticulous cartography of the myocardium to pinpoint the most optimal locations for injection, circumventing regions of total tissue death and instead concentrating on the transitional areas between ischemia and unaffected tissue. The stem cells were administered in multiple small doses across these areas to achieve a uniform distribution and reduce the likelihood of local tissue disruption. The EVs were co-transplanted with the stem cells to amplify the paracrine signaling effects, given their aptitude to mediate the microenvironment and support the healing. The main objective of this intervention was to stabilize the patient’s cardiac function and initiate a regenerative process aimed at improving ventricular performance progressively. Our approach of combining CABG with regenerative therapy was center on the intent to restore blood flow to ischemic myocardium and to promote the repair and regeneration of damaged myocardial tissue, leading towards improved EF and decreased ventricular volumes.

### Post-Operative Course and Follow-Up

Following the CABG procedure, the patient was transferred to the intensive care unit (ICU) for comprehensive monitoring. The initial post-operative course was uneventful, with stable hemodynamics supported by the IABP. However, on post-operative Day 3, the patient developed sudden ventricular arrhythmia and experienced a cardiac arrest. Prompt cardiopulmonary resuscitation (CPR) was performed, and the patient was successfully stabilized with anti-arrhythmic therapy.

Serial follow-up imaging studies were conducted to monitor graft patency and myocardial recovery. One month after the surgery and cell therapy, MPS and ECHO assessments showed a slight decline in cardiac function. EF decreased from 28 to 24%, EDV increased from 182 to 184 ml, and ESV rose from 132 to 140 ml (Figs. 2B and 4). Despite optimized medical therapy—including beta-blockers, ACE inhibitors, and statins—early functional response was limited.

**Fig. 3:**
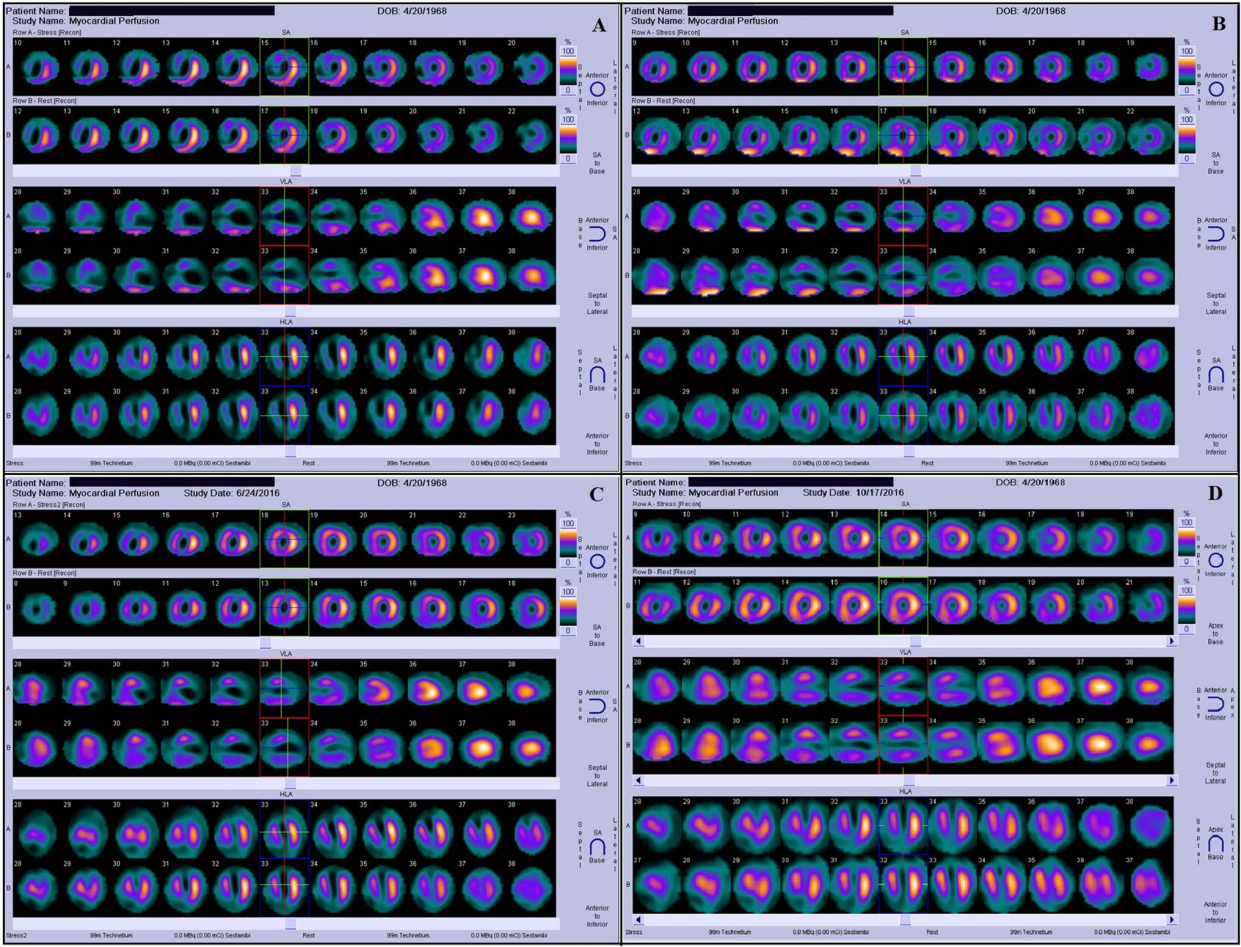
Myocardial perfusion images (stress and rest states) are shown in short-axis (SA), vertical long-axis (VLA), and horizontal long-axis (HLA) views at four different time points: **A** Initial Evaluation (Pre-op 1 month): Baseline perfusion imaging reveals multiple areas of reduced perfusion, particularly during stress testing. These darkened regions represent significant perfusion defects, indicating compromised blood flow and ischemia. **B** Post-op 1 month: There is minimal improvement in myocardial perfusion during both stress and rest states. Perfusion defects remain evident, with persistent areas of ischemia. **C** Post-op 3 months: Significant improvement in perfusion is observed. There is a marked reduction in perfusion defects, with more consistent blood flow during both stress and rest conditions. **D** Post-op 6 months: The final follow-up demonstrates sustained and substantial improvement in myocardial perfusion. The ischemic areas seen previously are significantly diminished, with no new perfusion defects. The imaging reflects stable and enhanced blood flow, highlighting the long-term impact of MSC therapy on myocardial recovery and function

**Fig. 4:**
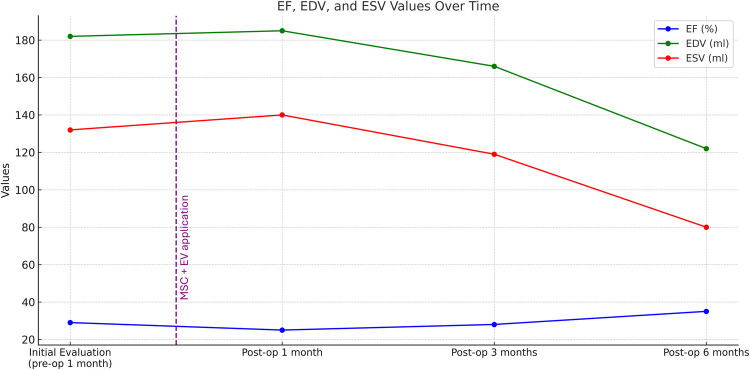
Changes in cardiac parameters over time following MSC + EV therapy. This graph illustrates the temporal changes in key cardiac parameters, including EF, EDV, and ESV, in a patient undergoing MSC + EV therapy. The time of cell therapy administration is marked by a vertical dashed line on the graph. Ejection Fraction (EF %): The EF showed a delayed but steady improvement over time, starting at 28% during the initial evaluation (pre-op 1 month). There was a slight decrease to 24% at 1 month post-op, followed by an increase to 28% at 3 months post-op, and further improvement to 35% at 6 months post-op, indicating a gradual enhancement in myocardial contractility. End-Diastolic Volume (EDV, ml): The EDV showed a progressive decrease from 182 ml at the initial evaluation to 122 ml at 6 months post-op. This reduction in EDV suggests improved cardiac efficiency and a normalization of ventricular size over time. End-Systolic Volume (ESV, ml): Similarly, the ESV decreased from 132 ml pre-op to 80 ml at 6 months post-op, indicating more effective ejection of blood with each heartbeat as the therapy took effect. These trends collectively demonstrate the positive impact of MSC therapy on cardiac function, with gradual improvements in both the structural and functional parameters of the heart over time

At the three-month follow-up, cardiac function began to improve. ECHO showed an EF of 28%, with EDV decreasing to 166 ml and ESV to 119 ml (Figs. 2C and 4). Myocardial perfusion imaging revealed better perfusion in the previously ischemic anterior and septal walls, with visible recovery in wall motion (Fig. 3C). The patient’s functional capacity also improved to NYHA Class II, reflecting reduced symptom burden and improved quality of life.

At six months post-operation, repeat coronary angiography was performed due to the recurrence of exertional dyspnea. The imaging revealed that the bypass graft to LAD artery was occluded. Remarkably, despite the failed graft, ECHO demonstrated further improvement in cardiac function, with EF reaching 43%, EDV 136 ml, and ESV 78 ml (Figs. 1B, 2D, and 4). These results suggest a sustained regenerative response beyond the mechanical revascularization alone, likely attributed to the biologic effects of the combined stem cell and EV therapy. A concise timeline of the clinical course is provided below:

- Day 0: Off-pump CABG performed with direct intramyocardial injection of 33 × 10⁶ hUC-MSCs and 5 × 10⁹ EVs.
- Day 3: Ventricular arrhythmia and cardiac arrest; resuscitated and stabilized.
- 1 Month Post-op: EF dropped to 24%, with slight increases in EDV and ESV.
- 3 Months Post-op: EF improved to 28%, perfusion and wall motion began to recover.
- 6 Months Post-op: EF reached 43% (ECHO) and 35% (MPS), with improved myocardial function despite LAD graft occlusion.

### Clinical Implications

Especially in patients with failed revascularization or high-risk surgical profiles, this case underscores the potential value of combining MSC and EV therapy as a meaningful adjunct to conventional cardiac surgery. Despite early postoperative deterioration and eventual graft occlusion, the sustained improvement in cardiac function suggests that intramyocardial delivery of regenerative agents may activate intrinsic repair mechanisms that persist independently of graft patency. These findings support the integration of biologic therapies into standard surgical protocols and highlight the need for further research to establish safety, efficacy, and optimal delivery strategies in the treatment of acute myocardial infarction.

## Discussion

The results of this case report align with previous preclinical findings regarding the therapeutic potential of EVs and MSCs in myocardial regeneration. Animal studies have demonstrated that EVs stimulate healing in infarcted myocardium [6], prevent HF progression [7], and mediate these effects primarily through paracrine signaling mechanisms. The intramyocardial injection of EVs and MSCs during the bypass surgery in this case aimed to enhance direct repair of ischemic regions. ECHO and MPS evaluations during our regular follow-ups demonstrated a significant increase in EF and improved wall motion in the anterior wall, even after the occlusion of the bypass graft (Figs. 1, 2, 3 and 4). Typically, permanently akinetic regions would be expected post-infarction, highlighting the potential regenerative impact of the therapy.

Following MI, several cytokines and growth factors initiate inflammatory mechanisms to promote tissue regeneration. Several studies indicate that acute cardiac damage triggers the activation and replenishment of both cardiac progenitor cells and the reprogramming of non-cardiac progenitor cells from the bone marrow to the site of injury [8–10]. However, clinical research has shown that bone marrow- or blood-derived stem cells do not significantly regenerate cardiac tissue. Instead, MSC-derived EVs are recognized as the primary mediators of cardiac repair [11]. EVs facilitate healing via miRNA transfer, promoting anti-inflammatory and anti-fibrotic responses [7, 12–14]. Importantly, EVs produced by different stem cell types carry distinct miRNA profiles, influencing their regenerative effects [15–18]. Hence, EVs produced from distinct stem cells are not identical, since each EV carries the specific miRNA profile of its parent stem cell phenotype.

In our case, we derived EVs from allogenic hUC-MSCs. Preclinical studies suggest that hUC-MSC derived EVs are a safe and effective cell-free strategy to promote angiogenesis [19, 20]. Compared to autologous stem cells, hUC-MSCs offer advantages such as superior immunomodulation, ease of extraction, and faster proliferation [20, 21]. Furthermore, MSC-derived EVs have been shown to improve cardiac stem cell survival and function [21]. Although no adverse effects were observed in this case, we acknowledge reports of rare complications such as pulmonary embolism following high-dose EV administration [22].

In our approach, EVs and MSCs were injected directly into the border zone between infarcted and viable myocardium [18, 23], an area known to exhibit ischemia without necrosis. The timing and method of delivery were chosen based on evidence that direct intramyocardial injection during surgery provides superior tissue targeting and regenerative stimulus [24, 25].

In patients undergoing CABG, improvements in EF are typically modest. Depending on baseline function and viability studies [26–28] indicate increases ranging from ~ 3% to 10%. Soetisna et al. observed an average EF improvement of + 2.91% [26]; Koene et al. reported an increase from 36 to 41% [27]; Ponuru et al. highlighted an improvement from 29.5% to 39.9% [28]. Although no causal conclusion can be made in a single-patient example, our patient's EF increased from 25 to 45%, suggesting a possibly enhanced regeneration impact beyond typical CABG recovery. Therapeutic use of MSCs and MSC-derived EVs in cardiovascular disorders has been investigated extensively [29, 30]. MSCs contribute to myocardial recovery primarily through paracrine mechanisms, including modulation of inflammation, promotion of angiogenesis, and attenuation of fibrosis [30]. EVs produced from MSCs have shown promise in improving heart function, lowering infarct size, and protecting against ischemia–reperfusion damage [31]. Although most past research has focused on systemic or intracoronary administration after infarction, integration of regenerative treatments during cardiac operations is attracting increasing attention. Our case enhances the growing corpus of data. In our case, MSC and EV application to the border zone of the infarcted area was performed during bypass revascularization. After a short period of time, the patient experienced occlusion of the bypass graft in the LAD territory. Despite failed revascularization during subsequent PCI attempts, the healing process continued, suggesting that EV-mediated effects may have supported tissue repair even under hypoxic conditions [32, 33].

Limitations: This report represents a single-patient observation, limiting generalizability. No control group or comparator was available. Mechanistic assessments, including direct markers of angiogenesis, fibrosis, or inflammation (e.g., VEGF-A, hs-CRP, IL-6, TNF-α), were not performed. While clinical follow-up showed no adverse effects, the absence of systemic biomarker monitoring is acknowledged. Future studies involving larger cohorts, molecular analyses, and prolonged follow-up are required to validate and expand these findings.

## Conclusion

The field of cell therapy and research on EVs in cardiovascular medicine is fast expanding; however, many questions are still left unaddressed. The ultimate objective is to optimize methods for isolation of EVs and for targeted delivery to the heart. This will allow for safe therapeutic use for cardiovascular disease. EV-based cell-free therapy represents a promising adjunct to revascularization strategies in treating acute and subacute MI. Such an approach could potentially prevent the progression to HF or improve myocardial healing with or without revascularization. Not all MI patients are suitable candidates for revascularization, and even revascularization success, ischemia–reperfusion injury can compromise cardiac recovery.

Preclinical studies have shown that EVs provide protective effects in conditions involving ischemia–reperfusion injury. In the present case report, we observed remarkable improvements in cardiac function following the administration of EVs and MSCs during a CABG procedure. ECHO and MPS were used to monitor these improvements in myocardial wall motion. Future clinical studies could benefit from incorporating advanced imaging techniques such as cardiac MRI or PET scanning. These modalities offer comprehensive insights into myocardial viability and stages of healing, potentially offering a more comprehensive assessment than ECHO and MPS alone.

In recent years, studies have increasingly focused on the potential of EV-based therapies in cardiovascular diseases, shifting the focus from traditional cell therapy to cell-free approaches. While the benefits of EV therapy are promising, significant challenges and limitations, such as scalability and delivery methods, require attention. More clinical studies are required in order to better comprehend and exploit the therapeutic feasibility of EVs for heart diseases. In conclusion, while our case underscores the potential benefits of combining stem cells and EVs in treating myocardial infarction, continuous research and clinical trials are crucial to fully understand their therapeutic impact and enhance their clinical application.

## Data Availability

The datasets generated and analyzed during the current study are available from the corresponding author upon reasonable request.
